# Enzymatic activity of palmitoyl‐protein thioesterase‐1 in serum from schizophrenia significantly associates with schizophrenia diagnosis scales

**DOI:** 10.1111/jcmm.14496

**Published:** 2019-07-03

**Authors:** Yaoyao Wu, Qianqian Zhang, Yawei Qi, Jingjing Gao, Wenqiang Li, Luxiang Lv, Guanjie Chen, Zhongjian Zhang, Xuyi Yue, Shiyong Peng

**Affiliations:** ^1^ Section on Molecular Imaging and Signal Transmission (MIST), Institute of Psychiatry and Neuroscience (IPN) XXMU Xinxiang China; ^2^ Henan Key Lab of Biological Psychiatry The Second Affiliated Hospital of Xinxiang Medical University Xinxiang China; ^3^ International Joint Research Laboratory for Psychiatry and Neuroscience of Henan Henan Mental Hospital Xinxiang China; ^4^ National Human Genome Research Institute (NHGRI), NIH Bethesda Maryland; ^5^ Section on Developmental Genetics PDEGEN, NICHD, NIH Bethesda Maryland

**Keywords:** biomarker, enzymatic activity, palmitoyl‐protein thioesterase, schizophrenia

## Abstract

Genome‐wide association studies have confirmed that schizophrenia is an inheritable multiple‐gene mental disorder. Longitudinal studies about depression, first episode psychosis (FEP) and acute psychotic relapse have mostly searched for brain imaging biomarkers and inflammatory markers from the blood. However, to the best of our knowledge, the association between enzymatic activities with diagnosis or prediction of treatment response in people with schizophrenia has barely been validated. Under the Longitudinal Study of National Mental Health Work Plan (2015‐2020), we have studied a subsample of approximately 36 individuals from the cohort with data on palmitoyl‐protein thioesterase‐1 enzymatic activity from FEP and performed a bivariate correlation analysis with psychiatric assessment scores. After adjusting for sex, age, body mass index (BMI) and total serum protein, our data demonstrated that PPT1 enzymatic activity is significantly associated with schizophrenia and its Positive and Negative Syndrome Scale (PANSS) scores. This longitudinal study compared the PPT1 enzymatic activity in FEP schizophrenia patients and healthy volunteers, and the former exhibited a significant 1.5‐fold increase in PPT1 enzymatic levels (1.79 mmol/L/h/mL, and 1.18 mmol/L/h/mL; *P* < 0.05; 95% CI, 2.3‐2.9 and 1.4‐1.8). The higher PPT1 enzymatic levels in FEP schizophrenia patients were positively associated with larger PANSS scaling scores (*r* = 0.32, *P* = 0.0079 for positive scaling; *r* = 0.41, *P* = 0.0006 for negative scaling; *r* = 0.45, *P* = 0.0001 for general scaling; and *r* = 0.34, *P* = 0.0048 for PNASS‐S scaling). Higher enzymatic PPT1 in FEP schizophrenia patients is significantly associated with increased PANSS scaling values, indicating more serious rates of developing psychosis. Enzymatic activity of PPT1 may provide an important new view for schizophrenia disorders.

## 1 INTRODUCTION

Schizophrenia (SCZ) is a mental illness that is characterized by abnormal social behaviour and misunderstanding of reality.^1^, ^2^, ^3^ Nearly 1% of the world's population will be affected by schizophrenia in their lifetime.^4^, ^5^, ^6^ In 2016, there were an estimated 21 million people with schizophrenia,^7^ in which men were more often affected by schizophrenia than women.^8^ The average life expectancy of people with schizophrenia is 10‐25 years lower than the average life expectancy.^9^, ^10^ The reason behind this lower life expectancy is that patients with schizophrenia have higher rates of physical health problems and higher rates of suicide.^9^ In 2015, 17,000 people died from schizophrenia or suicide worldwide.^11^, ^12^ The causes of schizophrenia include environmental and genetic factors.^13^, ^14^ Possible environmental factors include the use of recreational drugs, certain infectious diseases, parental age, and inadequate nutrition in the mother's womb.^15^ In SCZ diagnosis, the cultural background of the subjects must be taken into consideration.^16^ So far, there are no objective factors for biological diagnostic criteria 16.

After protein translation, the protein structure undergoes lipid modification and becomes more complex and more functional, which plays a vital role in the organism and maintains regular synapse connection in the brain.^17^ Palmitate is the most abundant fatty acid in the organism and can be covalently bonded to proteins.^17^, ^18^ According to the connection site, palmitoylation can be divided into n‐type and s‐type palmitoylation (via an amide linkage to glycine and cysteine residues).^19^ S‐palm acylating modification (S‐palmitoylation) refers to the 16 carbon situated palmitate through sulphur ester covalent linking to the protein cysteine thiol amino acid residue, which is currently the only known form of dynamic, reversible modification after translation.^20^, ^21^ Palmitoylated modification controls the function of synaptic proteins temporally and spatially.^17^ Soluble palmitoylated proteins, such as signalling proteins, adhesion molecules and neurotransmitter receptors, regulate the activity and stability of proteins and protein‐protein interactions within a synapse whose abnormal functions of transmission and plasticity are hallmarks in the schizophrenic brain.^20^, ^21^, ^22^ Moreover, dynamic palmitoylation plays an essential role in cell signalling, metabolism, apoptosis and mental illness.^17^, ^22^

Dynamic palmitoylation is manipulated by reciprocal enzymatic activities performed by palmitoyl acyltransferase (PAT) and palmitoyl‐protein thioesterase (PPT).^17^, ^23^ PPTs are depalmitoylated enzymes consisting of PPT1 and PPT2, mainly in living organisms.^24^ Palmitoyl‐protein thioesterase 1 is encoded by ceroid lipofuscinosis neuronal‐1 gene whose mutation causes the infantile neuronal ceroid lipofuscinosis, a group of the five most prevalent (1 in 12,500 births) neurodegenerative lysosomal storage disorders.^25^, ^26^ Most importantly, PPT1 is currently identified as one of the genetic risk variants through a genome‐wide association study (GWAS) analysis.^14^, ^27^, ^28^ Dynamic palmitoylation by PATs and PPT1 can regulate the stability, localization, and function of many receptors on neuronal synapses, playing functional roles and engaging their respective ligands for canonical synaptic connections, which are aberrantly wired in schizophrenic brains.^17^, ^22^ Moreover, dynamic palmitoylation plays essential roles in developing brains.^23^ Our previous study has demonstrated that PPT1 deficiency causes synaptic dysfunction and dendritic loss in the very early developing mouse brain.^25^ Interestingly, Ibrahim and his colleagues 28 analysed transcriptional expression of 33 candidate genes and showed that several blood‐based markers, including the PPT1 gene, were weakly correlated with functional magnetic resonance imaging in specific brain regions in SCZ compared to those matched healthy volunteers. Indeed, a PPT1 mimetic, NtBHA, could also prolong the life span and brain functions in a neuronal dysfunction animal model.^29^, ^30^

Using a subsample from the National Mental Health Work Plan (2015‐2020), a treatment cohort study based in Xinxiang City, Henan, China,^31^, ^32^ we hypothesized that the outside enzymatic activity of PPT1 could be a sign for diagnosing or predicting the progression of schizophrenia in clinical settings. In this study, circulating PPT1 levels in the blood were investigated from schizophrenia patients and matched to healthy volunteer subjects. Notably, we utilized initially a commercial protocol that could sensitively detect the enzymatic activity of PPT1 in blood. Our results demonstrated that enzymatic PPT1 activity in blood from patients with SCZ is significantly more potent than that of the healthy volunteers. Considering study design, the distributions of age and sex showed no difference between SCZ patients and controls. Body mass index (BMI) and total serum protein in SCZ patients were significantly higher than in matched controls. Pearson correlation scores between the PPT1 enzymatic activity and transformed PNASS scales were significantly correlated to PNASS scales. Our results suggest that mental dysfunction of SCZ in the central nervous system could be examined through peripheral blood circulating factors, such as PPT1 enzymatic activity. The current study sheds light on distinguishing schizophrenia complexes in the future.

## 2 METHODS

### 2.1 Description of patients and recruitment process

This study used a subsample from the National Mental Health Work Plan (2015‐2020), a treatment cohort study based in Xinxiang City, Henan, China.^32^ All outpatients and inpatients were diagnosed with positive, negative, and cognitive impairment or emotional syndrome, according to the PNASS factor scores. Inclusion criteria: (a) age 18‐60 years old; (b) no infectious disease; (c) no chronic cardiovascular, digestive tract, endocrine, immune system and/or respiratory diseases within 2 weeks before blood sampling; (d) no family history of diabetes and no primary liver and kidney disease; (e) no bad habits, such as addiction and alcohol abuse; (f) not pregnant or lactating; (g) could be treated with mood stabilizers, atypical antipsychotics but not with antihypertensive drugs and typical antipsychotics; and (h) provided informed consent. The positive and negative syndrome scale (PANSS) 6 scaling system was used to define the severity of patients with schizophrenia based on the interview reports of family members or primary care hospital workers. Cases of schizophrenia were defined 33 and 31 cases of matched healthy volunteers were recruited for this current study. This clinical study was approved by the Second Affiliated Hospital Ethics Committee of Xinxiang Medical University (XXMU).

### 2.2 Laboratory methods

Blood samples (5 mL/each) from both healthy volunteers and patients after overnight fasting were collected in a test tube containing EDTAK2 anticoagulant. After centrifuging at 1000 rpm for 10 minutes, the plasma was stored at −80°C for later use.

Before electrophoresis or ELISA assays, each serum sample was treated with a Qproteome Albumin/IgG Depletion Kit (Sigma, Shanghai, China). For western blotting, primary antibodies were as followed: rabbit anti‐PPT1 (ab38417, 1:1000, Abcam, Shanghai, China); rabbit anti‐transferrin (ab137744, 1:2000, Abcam, Shanghai, China). The secondary antibody was goat anti‐rabbit IgG (sc‐2054, 1:1500, Santa Cruz Biotechnology Inc, Dallas, TX.). The PPT1 enzymatic assay (Moscerdam Substrates, 2341 KS Oegstgeest, the Netherlands) was performed after 8 months with no freeze‐thaw cycles during storage. All intra‐assay coefficiency of variations was less than 5%. Please also see in supplemental materials.

### 2.3 Psychiatric measures: clinical evaluation in schizophrenia patients

Details of the clinical evaluation in the schizophrenia patients have been published elsewhere.^32^ The patients with positive symptoms had scores from 14 to 28. The patients with negative symptoms had scores from 13 to 30, and the patients with general symptoms had scores from 38 to 52.

### 2.4 Statistical analysis

The PANSS 6 was used to measure symptom severity of patients with schizophrenia. Thirty different symptoms based on the interview reports of family members or primary care hospital workers were rated from 1 to 7. Totals of 7, 7 and 16 symptoms relate to positive, negative and general scales respectively. The minimum score for positive, negative and general scale is 7, 7 and 16, and is used for matched healthy controls. The Student's *t* test was used to compare two groups to continuous variables. Chi‐square test was used to compare two groups in binary variables. PANSS scales (positive, negative and general) and S‐scales were regressed on age, sex, BMI, serum total protein levels. The resulting residuals were ranked and inverse normalized. The multiple linear regressions in SAS 9.14 were used for analyses of the transformed PANSS scales. The logistic regression model was used for the binary variable of FEP status with covariates of sex, age, BMI and serum total protein level. The Pearson correlation coefficient scores were calculated among PPT1, sex, age, BMI and serum total protein, regressed, ranked and inverse normalized PNASS scales. Receiver operator characteristic (ROC) curves analysis in R (pROC) was performed to evaluate if PPT1 activity was a predictor of SCZ. The data set was randomly divided into train (60%) and test (40%) sets. Parameters were estimated in a train set with covariant of sex, age, BMI and serum total protein, and the parameters were evaluated in test set. ROC and AUC (area under a curve) scores were calculated based on the predicted probabilities within a test set. *P*< 0.05 was considered statistically significant.

## 3 RESULTS

Demographic and clinical details of the samples are presented in Table 1. There was no difference in sex and age between schizophrenia patients and matched healthy volunteer groups. The BMI and serum protein and enzymatic activities of PPT1 levels in the SZ group were significantly higher than those from the healthy volunteer group with *P* < 0.01**.**

**Table 1:** Study information

	SZ	NC	*P* values^a^
N	36	31	
Sex (male, %)	18 (50%)	21 (68%)	0.3541
Age (y)	28.1 (8.9)	28.7 (6.6)	0.7642
BMI (kg/m^2^)	24.0 (4.4)	21.4 (2.2)	0.0022
Serum protein (mg/mL)	6.4 (0.9)	4.9 (1.6)	<0.0001
PPT1 (mmol/L/h/mL)	1.79 (0.09)	1.18 (0.06)	<0.0001
PANSS			
Positive scale	21.9 (6.3)		
Negative scale	22.1 (7.8)		
General scale	43.2 (9.1)		
S scale	8.1 (4.0)		

Note

Present: mean (SD).

Abbreviation: NC, healthy volunteer subjects; SZ, schizophrenia; PANSS, Positive and Negative Syndrome Scale; BMI, Body Max Index; PPT1, palmitoyl protein thioesterase‐1.

a Chi‐square test was used to compare two groups in binary variables.

### 3.1 Palmitoyl protein thioesterase‐1 was significantly increased in enzymatic activity in schizophrenia patients

Palmitoyl‐protein thioesterase 1 was weakly correlated with functional magnetic resonance imaging in specific brain regions,^28^ and the PPT1 gene was analysed as one of the candidate genes showing potential for a blood‐based marker in SCZ compared to those matched healthy volunteers. Our data demonstrated that PPT1 immunoreactive signals from SCZ patients’ blood were no different between compared to those from healthy volunteers (Figure S1). We then sought to detect the enzymatic activity of PPT1. Interestingly, our results showed that the enzymatic activity of PPT1 from SCZ patients (1.79 mmol/L/h/mL) is significantly increased by 1.5‐fold compared with that from healthy volunteers (1.18 mmol/L/h/mL) (Table 1 and Figure S2B,C).

### 3.2 Enzymatic activity levels of palmitoyl protein thioesterase‐1 in serum were highly prone to predict the severity of schizophrenia or schizophrenia spectrum disorders

From the current subsample of the longitudinal study, the distributions of age and sex were not different between the SCZ patients and matched healthy volunteer subjects as a study designed. Basic study information is presented in Table 1.

Body mass index, total serum protein and PPT1 enzymatic value in the SCZ patients were significantly higher than those in the matched controls (Table 1). Pearson correlation scores between the PPT1 enzymatic activity levels and the transformed PNASS scales are presented in Table 2 and showed that the PPT1 enzymatic activity levels were significantly correlated with the PNASS scales with *P* < 0.01 (*H*_0_; Partial *ρ* = 0).

**Table 2:** Pearson correlations in Schizophrenia cases

	Positive	Negative	General	S score^a^
PPT1	0.32 0.0079	0.41 0.0006	0.45 0.0001	0.34 0.0048
Positive^a^		N/A	0.90 <0.0001	N/A
Negative^a^			0.81 <0.0001	N/A
General^a^				0.87 <0.0001

*P* values under *H*_0_: partial *ρ* = 0, regressed by sex, age, BMI, and protein. N/A, not applicable

a Presents Pearson partial correlation coefficients.

The associations between PPT1 enzymatic activity levels and scaling scores of schizophrenia with and without covariate of serum total protein were evaluated further. Our results showed that the PPT1 enzymatic activity levels were significantly associated with SCZ status with odd ratios 6.19 (95% CI: 2.23, 17.25) and 9.51 (95% CI: 2.39, 37.83) for with and without adjustment of total serum protein levels respectively (Table 3).

**Table 3:** Association results

Model	Response^a^	PPT1					
		Beta	SE	*P* values	ORS (95% CI)	Covariates	Adj. R2
Logistic regression	SCZ	1.82	0.52	0.0005	6.19 (2.23, 17.25)	Age, sex, BMI	
	SCZ	2.25	0.7	0.0014	9.51 (2.39, 37.83)	Age, sex, BMI and protein	
Regression	Positive^b^	0.36	0.13	0.0079			0.09
	Negative	0.46	0.13	0.0006			0.15
	General	0.51	0.12	0.0001			0.19
	S score	0.38	0.13	0.0048			0.1

a SCZ: Binary variable

b Regressed in sex, age, BMI and serum total protein, then ranked and inverse normalized PANSS scales

Most importantly, higher levels of blood‐based PTT1 enzymatic activity were significantly associated with higher PNASS scales with *P*< 0.01, and explain 9%, 15%, 19% and 10% variances of positive, negative, general and S scales respectively (Table 3).

**Figure 1:**
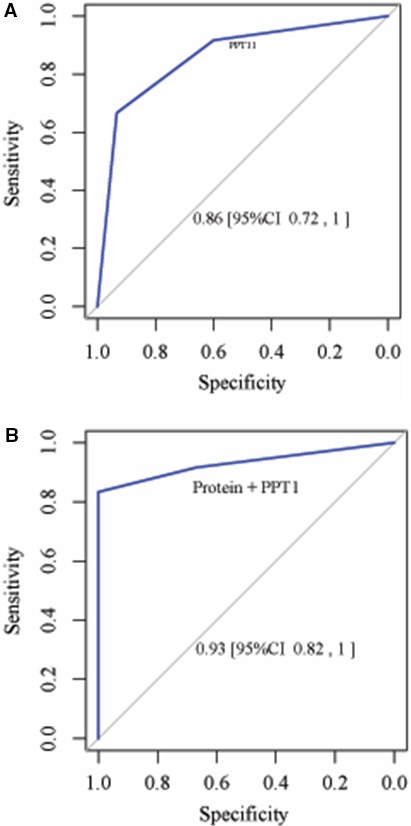
Receiver operator characteristic (ROC) curves and area under a curve (AUC) analysis. left The ROC curves were determined with the PPT1 enzymatic activity only; right The AUC scores were for the PPT1 enzymatic activity with adjusted serum total protein levels

The schizophrenia group had higher PPT1 enzymatic activities and serum total protein levels than the control group. The PPT1 enzymatic activity was shown to be significantly associated with SCZ status and PNASS scaling scores in this longitudinal study. To determine whether blood‐based PPT1 enzymatic activity can be applied as a diagnostic biomarker in schizophrenia or schizophrenia spectrum disorders, we performed ROC curves analysis. We determined the ROC and AUC (area under a curve) scores for PPT1 enzymatic activity only (Figure 1A) and PPT1 enzymatic activity with adjusted serum total protein level (Figure 1B). Our results demonstrated that the PPT1 enzymatic activity with adjustment of serum total protein was a better biomarker with an AUC 0.96 (95% CI, 0.9, 1).

## 4 DISCUSSION

Our findings demonstrated, in a longitudinal cohort study, that PPT1 enzymatic activity levels associated with FEP schizophrenia clinical assessment scores. This association persisted after adjusting for several potential confounders, including sex, age, BMI and total serum protein levels. Higher PPT1 enzymatic activity levels were extremely positively correlated with FEP schizophrenia with higher PANSS‐positive scaling scores (*r* = 0.32, *P* = 0.0079), negative scaling scores (*r* = 0.41, *P* = 0.0006), general scaling scores (*r* = 0.45, *P* = 0.0001) and PNASS‐S scaling scores (*r* = 0.34, *P* = 0.0048).

To the best of our knowledge, this is the first longitudinal study of enzymatic activity levels on blood‐based assay in first episode patients of schizophrenia. We applied restricted categories of psychotic measurements to assess each FEP patient and the degree of severity according to the latest version of the PANSS scaling system.^6^, ^7^ Previously, distinct profiles of inflammatory factors, oxidative stress elements and changes of immune cell counts^31^, ^34^, ^35^ were highly studied and inconsistent. Conflicting results were likely due to the immune process, which is highly dynamic and changes in which are often transient. Higher levels of blood glutamate in SCZ were found and had potential to act as a diagnostic marker for SCZ,^36^ but it correlated with major depressive disorder.^33^ Oxidative markers are also examined in some studies, but oxidant markers have strong overlapping levels between people with SCZ and healthy individuals.^37^, ^38^ In our current longitudinal study, data from the serum levels of inflammatory factors and oxidant elements were not feasibly detected, although we found that serum levels of brain‐derived neurotrophic factor (BDNF) were substantially increased in FEP SCZ patients compared to those in healthy volunteers (*P* < 0.05, Figure S3). These confronted results of serum factors in oxidative and immune profiles will limit their value to determine whether oxidative and inflammatory markers as good candidates for diagnosing or indicating treatment responses. However, the enzymatic activity of PPT1 in the current study provides a potentially novel path for seeking diagnostic serum biomarkers of schizophrenia.

Schizophrenia alters basic brain processes of perception, emotion and judgement to cause hallucinations, delusions, thought disorder and cognitive deficits.^8^ Through comprehensive GWAS from siblings of patients with SCZ, researchers have identified more than 100 susceptibility genes.^39^

Of all these related genes, PPT1 is one of the responsive genes that encode critical proteins for neural development, synaptic transmission and plasticity that are widely improperly expressed in the SCZ brain.^28^ A clearer understanding of the role of palmitoyl‐protein thioesterase‐1 in the pathogenesis of psychotic deterioration may lead to new treatments. Nt‐BHA, a PPT1 mimetic, could improve synaptic transmission properties and plasticity in our preclinical animal model study^29^, ^30^ encouraging the potential application for ameliorating synaptic dysfunction in SCZ conditions. Therefore, PPT1 enzymatic activity associated with the proceeding course of SCZ needs to be comprehensively examined in our future longitudinal study with larger observation scale. Nevertheless, preclinical research to identify specific PPT1 enzymatic pathways contributing to neuropsychiatric symptoms may help to devise more targeted interventions.

The limitations of our present study are that these FEP patients are still enrolled in longitudinal treatment studies and that the 90% dropout rate makes the follow‐up evaluation of serum levels of PPT1 enzymatic activity unavailable in the current study. However, associations between PPT1 enzymatic activity and psychotic assessment scores are consistent with the clinical and genetic studies from Ibrahim's group that 33 genes, including PPT1, as the potential blood‐based markers that are related to clinical fMRI changes in left hippocampal ventricle integrity. This view of the association of PPT1 enzymatic activity with severity PANSS scores provides a novel scope to the diagnosis or prediction of prognosis outcomes of schizophrenia in clinics.

## 5 CONCLUSIONS

The strong association between the systemic enzymatic levels of palmitoyl‐protein thioesterase‐1 in FEP schizophrenia patients and the psychotic assessment PANSS values could be used for diagnosing or predicting the severity of schizophrenia. Further study of PPT1 enzymatic activity in schizophrenia patients may provide crucial new intervention and prevention targets for schizophrenia disorders.

## ETHICAL APPROVAL AND INFORMED CONSENT

Uses of all human samples and blood tissues in this study complied with safety guidelines and regulations of the National Institute of Health (NIH). All experiments on human samples were approved by Xinxiang Medical University (XXMU) Ethics Committee.

## CONFLICT OF INTEREST

Authors of this paper claimed no potential financial and nonfinancial competing interest.

## Data Availability

Raw data were generated at Xinxiang Medical University. Derived data supporting the findings of this study are available from the corresponding author Peng, Shiyong on request.
