# Supplementary material for: Enzymatic activity of palmitoyl‐protein thioesterase‐1 in serum from schizophrenia significantly associates with schizophrenia diagnosis scales
Source: J Cell Mol Med. 2019 Jul 3;23(9):6512–8. doi: 10.1111/jcmm.14496 (PMC6714227; doi:10.1111/jcmm.14496)
Supplement: Supplementary file 1 [file JCMM-23-6512-s001.pdf]

Enzymatic activity of palmitoyl-protein thioesterase-1 in serum from schizophrenia significantly associates with schizophrenia diagnosis scales

Supplemental materials

Blood tissue preparation

Before electrophoresis or ELISA assay, each serum sample was treated with ProteoPrep® Immunoaffinity Albumin and IgG Depletion Kit (Sigma, PROTIA-1KT) to remove albumin and immune-globin proteins which are significant components in serum avoiding disturbing PPT1 movement in electrophoresis or preventing inflammatory and oxidative factors absorbance in ELISA experiments. Total levels of serum protein after purification was determined using the BCA assay (Beyotime, P0009). ELISA kits for BDNF, IL-8, TNF-α, and IFN-Y were purchased from ABclonal company (CatRK00433, RK00011, RK00033, RK00015 respectively). All ELISA results are shown in Figure 3.

Western blotting

Samples were separated on 12% sodium dodecyl sulfate–polyacrylamide gels at 120 V for 90 min as previously described [1]. Briefly, proteins were transferred onto polyvinylidene difluoride membranes in transfer buffer [25 mM Tris, 192 mM glycine, 20% (v/v) methanol] for 1.5 h at 120 Vat 4_C and blocked for 1 h at room temperature in Tris-buffered saline-Tween-20 [50 mM Tris, pH 8.0, 133 mM NaCl, 0.2% (v/v) Tween-20] with 10% (w/v) non-fat milk powder (BD, 232100). The blot was incubated with the anti-PPT1 antibody (1:1000, Abcam, ab38417), overnight at 4 ˚C in Tris-buffered saline-Tween-20. After washing in Tris-buffered saline-Tween-20, the membrane was incubated in horseradish peroxidase-conjugated goat anti-rabbit secondary antibodies (1 : 5000) in Tris-buffered saline-Tween-20 with 5% non-fat milk powder for 1 h at room temperature. Finally, an electrochemiluminescence system (Tanon, 080-501) was used to detect immunoreactive protein.

After PPT1 western blotting, each blot was reprobed with rabbit anti-transferrin (1 : 20 000, Abcam,ab137744) as the primary antibody and goat-anti-rabbit IgG as the secondary antibody for detection of the corresponding transferrin in each blood sample. Each sample was analyzed in triplicate in independent experiments. Representative blots for all samples are shown as in Figure 1.

Western blotting of serum PPT1 reactive signal shows a single band and similar density variations between schizophrenia and control groups

A

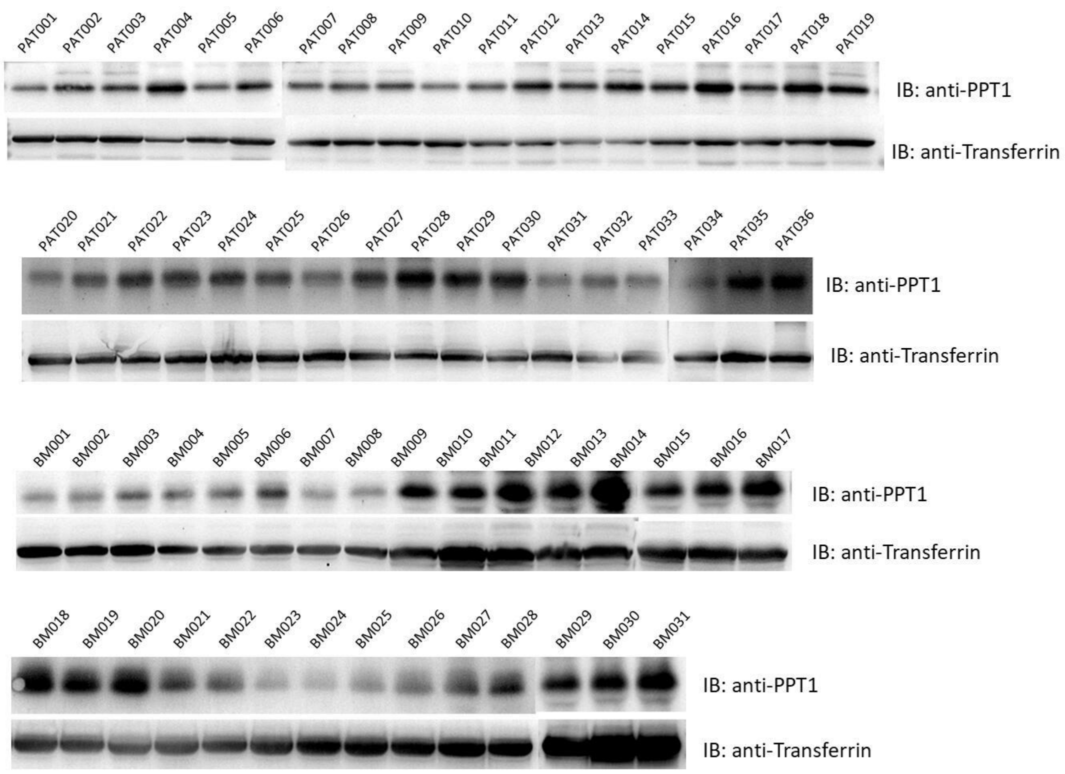

B

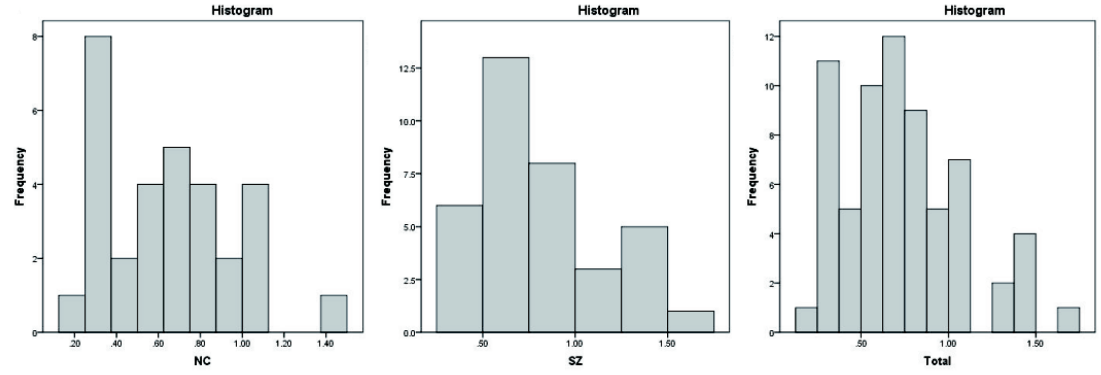

Figure 1. PPT1 in the serum of healthy subjects and patients with schizophrenia. A) Representative western-blot with anti-PPT1 and anti-Transferrin antibody to determine the abundance of PPT1 in healthy controls and schizophrenic patients. B) Histogram description the distribution of PPT1 protein level among all subjects, healthy volunteer group, and schizophrenia group. Kolmogorov-Smirnov test of PPT1 level among healthy volunteer, schizophrenia and Total, p=0.2*, 0.2*, 0.2*.

Enzymatic activity assay on PPT1 in blood samples

Before enzymatic assay, each serum sample was treated with ProteoPrep® Immunoaffinity Albumin and IgG Depletion Kit (Sigma, PROTIA-1KT) to remove albumin and immune-globin proteins which are significant components in serum avoiding interference with the accuracy of PPT1 enzymatic assay. 10 ul of each sample was used in this assay. The experiment was performed according to the manual instruction from the manufacturer of PPT1-substrate (Toronto research chemicals, M336675). The detailed procedures for measuring PPT1 enzymatic activity were also described in a previous fluorometric assay [2], and Figure 2A showed the standard curve. Briefly, in a 96-well ELISA plate 10ul of serum sample or 0.2% BSA (control) was mixed with 20 ul of substrate (3.4 mmol/L MU-6s-palm-bGlc in a 2:1 Chloroform: Methanol mixture). The plate was incubated at 37°C for 24 hours, and the reaction was terminated by adding 200 ul of stop solution (0.5 mol/L NaHCO3/0.5 mol/L Na2CO3 + 0.025% Triton X-100, pH 10). Ppt1 enzymatic activity was determined with absorbance wavelengths of 355 nm, λ_{ex} and 460 nm, λ_{em}. using a spectrofluorometer (TECAN, 1702001S). Activities were calculated according to the manual instructions of the substrate manufacturer, and the results were expressed as umol/ug/ul (eFigure 2B & C).

To role out the enzymatic activity of PPT1 associating with PPT1 protein levels in healthy and patients, the scatter plot between the blood PPT1 levels and the enzymatic activities of PPT1 was examined that there is no trend of correlations between PPT1 levels and the enzymatic activities of PPT1 (eFigure 2D).

A

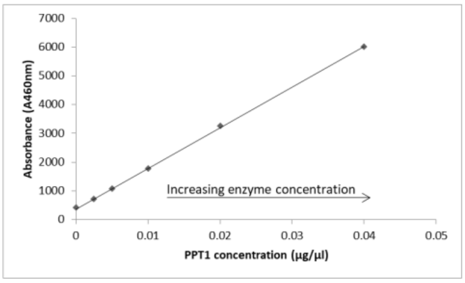

B

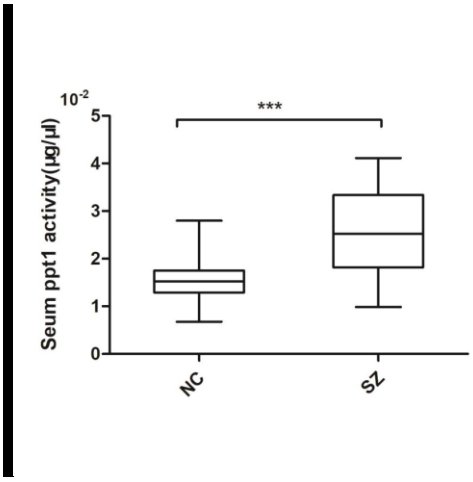

C

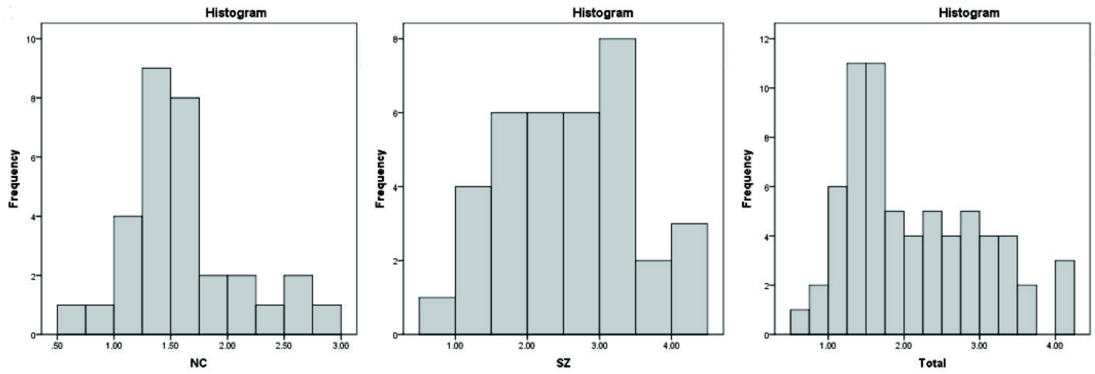

D

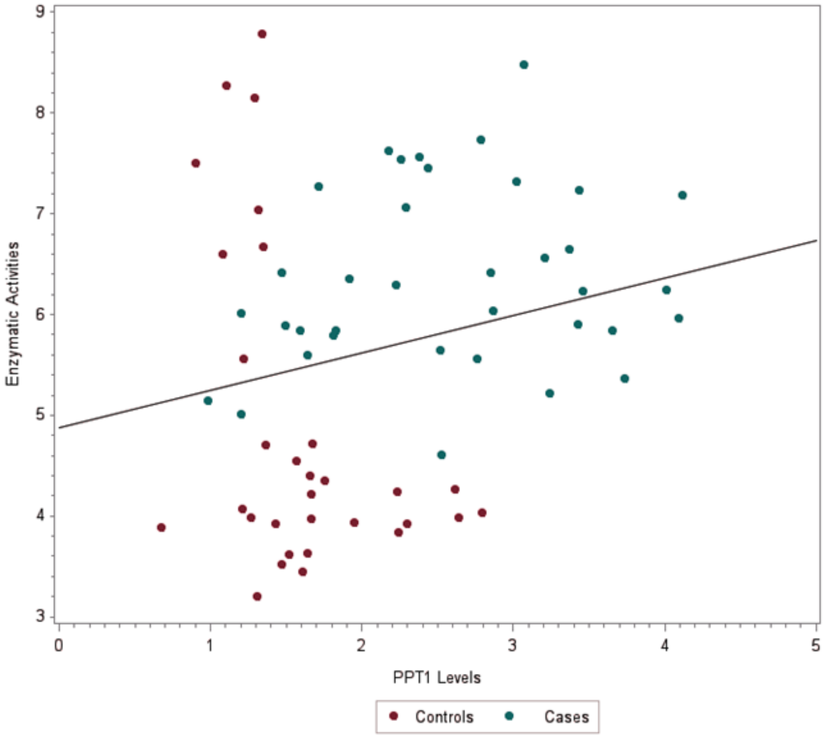

Figure 2. Enzymatic activity of PPT1 in the serum of healthy subjects and patients with schizophrenia. A) The standard curve of PPT1 enzymatic activity showing the absorbance of different concentrations of PPT1 standards. The X-axis is a sequential dilution of PPT1 standard; Y-axis is the measurement of absorbance at A460nm. Trend line formula: y=140921x+372.27, R=0.999. B) Box plot of serum PPT1 activity among healthy volunteer group and schizophrenia group. Serum PPT1 activity was calculated through the standard curve. C) Histogram description the distribution of PPT1 level among all subjects, healthy volunteer group, and schizophrenia group. Kolmogorov-Smirnov test of PPT1 level among healthy volunteer, schizophrenia and Total, p=0.2*, 0.06, 0.001. D). Scatter plot of serum PPT1 levels vs. Enzymatic activities of PPT1. X-axis presents serum PPT1 levels (ug/ul) and Y-axis presents Enzymatic activities of PPT1 (mM/hr/mL). Red dots present controls, and blue preset schizophrenia. The line presents the best fit line between PPT1 levels and enzymatic activity. The descriptive statistics of healthy volunteer: 1.521(0.46) (Median, Interquartile range); schizophrenia: 2.58±0.879 (Mean±SEM). Mann-Whitney U test was used for comparison, Z=-4.426, p=0.000. The significance was set as * P < 0.05; ** P < 0.01; *** P < 0.001.

There is no detectable difference between levels of IL-8, TNF-α, and IFN-Y, although serum brain-derived neurotrophic factor was significantly increased in schizophrenia

Previous studies postulated that pro-inflammatory factors, oxidative stress elements are potential blood biomarkers, although inconsistent results from different groups [3-5] . We have conducted ELISA experiments to detect certain inflammatory factors, IL-8, TNF-α and IFN-Y from our serum samples. Unfortunately, we could obtain marginal readings of IL-8, IL-1, and S100B, and did not draw any conclusive results from our ELISA data this time. A possible reason might be too much-diluted serum hardly to examine these factors, although the standard curves showed quite as sensitive detection of these inflammatory factors as ng/ml (Figure 3A). Another potential blood-based biomarker as previously postulated[6,7] was also tested in current serum samples. Our results demonstrated that there was a significant increase of serum levels of brain-derived neurotrophic factor （BDNF） in our SCZ patient sera compared to those in healthy volunteers (Figure 3B).

A

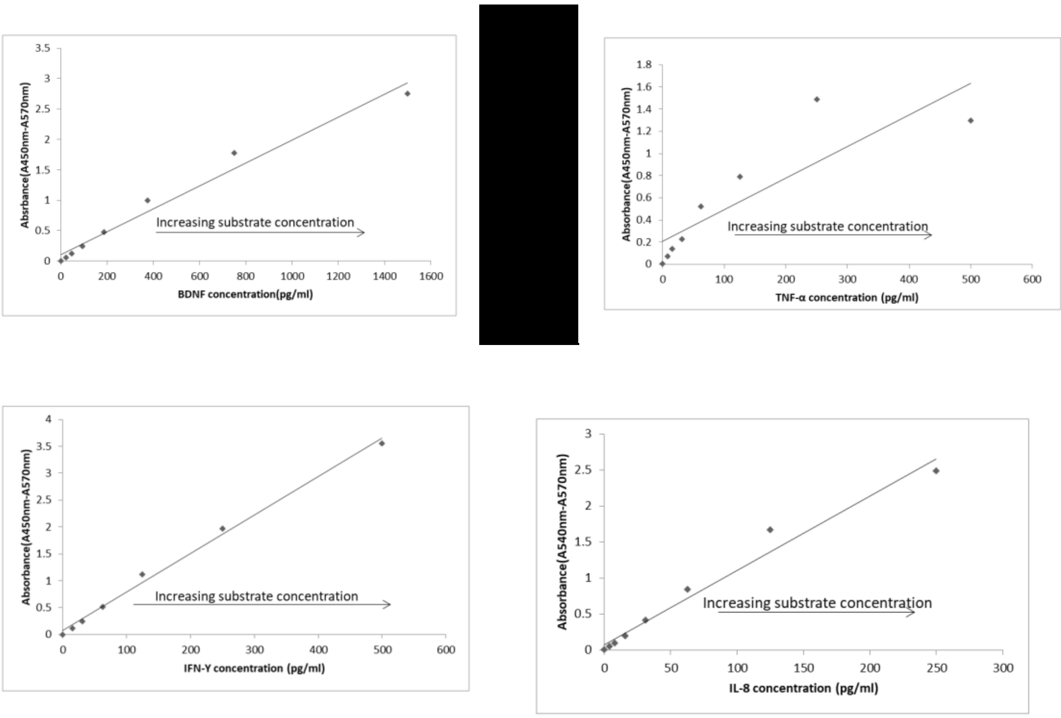

B

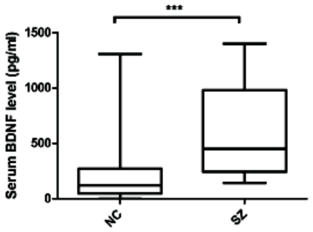

Figure 3. Quantitation of inflammatory factors: IL-8, TNF-α, IFN-Y, and brain-derived neurotrophic factor in the sera of healthy subjects and patients with schizophrenia. A) Standard curves of BDNF, TNF-α, IFN-Y, and IL-8 showing the absorbance of different concentrations of BDNF, TNF-α, IFN-Y and IL-8. The X-axis is sequential standard dilutions; Y-axis is the measurement of absorbance at A450nm minus absorbance at A570 nm. Trend line formula: BDNF $y=0.0019x+0.102$,$R=0.9883$; TNF-α $y=0.0029x+0.2088$, $R=0.8625$; IFN-Y $y=0.0072x+0.0687$, $R=0.9971$; IL-8 $y=0.0103x+0.0747$, $R=0.9871$. B) Box plot of serum BDNF level among healthy volunteer group and schizophrenia group. Serum BDNF level was calculated through the standard curve. The descriptive statistics of healthy volunteer: 121.632(220.73) (Median, Interquartile range); schizophrenia: 593.4±88.721 (Mean±SEM). Mann-Whitney U test was used for comparison, $Z=-3.625$, $p=0.000$. The significance was set as * $P < 0.05$; ** $P < 0.01$; *** $P < 0.001$.

References

1. **Peng S, Wuu J, Mufson EJ, Fahnestock M.** Precursor form of brain-derived neurotrophic factor and mature brain-derived neurotrophic factor are decreased in the pre-clinical stages of Alzheimer's disease. *Journal of neurochemistry*. 2005; 93: 1412-21.
2. **Jacks TJ, Kircher HW.** Fluorometric assay for the hydrolytic activity of lipase using fatty acyl esters of 4-methylumbelliferone. *Analytical biochemistry*. 1967; 21: 279-85.
3. **Ding M, Song X, Zhao J, Gao J, Li X, Yang G, Wang X, Harrington A, Fan X, Lv L.** Activation of Th17 cells in drug naive, first episode schizophrenia. *Progress in neuro-psychopharmacology & biological psychiatry*. 2014; 51: 78-82.
4. **Frydecka D, Krzystek-Korpacka M, Lubeiro A, Stramecki F, Stanczykiewicz B, Beszlej JA, Piotrowski P, Kotowicz K, Szewczuk-Boguslawska M, Pawlak-Adamska E, Misiak B.** Profiling inflammatory signatures of schizophrenia: A cross-sectional and meta-analysis study. *Brain, behavior, and immunity*. 2018; 71: 28-36.
5. **Leboyer M, Oliveira J, Tamouza R, Groc L.** Is it time for immunopsychiatry in psychotic disorders? *Psychopharmacology*. 2016; 233: 1651-60.
6. **Guillin O, Demily C, Thibaut F.** Brain-derived neurotrophic factor in schizophrenia and its relation with dopamine. *International review of neurobiology*. 2007; 78: 377-95.
7. **Pakhomova SA, Korovaitseva GI, Monchakovskaia M, Vil'ianov VB, Frolova LP, Kasparov SV, Kolesnichenko EV, Golimbet VE.** [Molecular-genetic study of early-onset schizophrenia]. *Zhurnal nevrologii i psikhiatrii imeni SS Korsakova*. 2010; 110: 66-9.
